# Genome-wide analysis and expression profiling of zinc finger homeodomain (*ZHD*) family genes reveal likely roles in organ development and stress responses in tomato

**DOI:** 10.1186/s12864-017-4082-y

**Published:** 2017-09-06

**Authors:** Khadiza Khatun, Ujjal Kumar Nath, Arif Hasan Khan Robin, Jong-In Park, Do-Jin Lee, Min-Bae Kim, Chang Kil Kim, Ki-Byung Lim, Ill Sup Nou, Mi-Young Chung

**Affiliations:** 1 Department of Agricultural Industry Economy and Education, Sunchon National University, 413 Jungangno, Suncheon, Jeonnam 57922 South Korea; 2 Department of Horticulture, Sunchon National University, 413 Jungangno, Suncheon, Jeonnam 57922 South Korea; 3 Department of Horticultural Science, Kyungpook National University, Daegu, 702-701 South Korea; 4 Department of Agricultural Education, Sunchon National University, 413 Jungangno, Suncheon, Jeonnam 57922 South Korea

**Keywords:** ZF-HD, *Solanum lycopersicum*, Phytohormone, Abiotic stress, Organ-specific expression, Fruit development

## Abstract

**Background:**

Zinc finger homeodomain proteins (ZHD) constitute a plant-specific transcription factor family with a conserved DNA binding homeodomain and a zinc finger motif. Members of the ZHD protein family play important roles in plant growth, development, and stress responses. Genome-wide characterization of *ZHD* genes has been carried out in several model plants, including *Arabidopsis thaliana* and *Oryza sativa*, but not yet in tomato (*Solanum lycopersicum*).

**Results:**

In this study, we performed the first comprehensive genome-wide characterization and expression profiling of the *ZHD* gene family in tomato (*Solanum lycopersicum*). We identified 22 *SlZHD* genes and classified them into six subfamilies based on phylogeny. The *SlZHD* genes were generally conserved in each subfamily, with minor variations in gene structure and motif distribution. The 22 *SlZHD* genes were distributed on six of the 12 tomato chromosomes, with segmental duplication detected in four genes. Analysis of Ka/Ks ratios revealed that the duplicated genes are under negative or purifying selection. Comprehensive expression analysis revealed that the *SlZHD* genes are widely expressed in various tissues, with most genes preferentially expressed in flower buds compared to other tissues. Moreover, many of the genes are responsive to abiotic stress and phytohormone treatment.

**Conclusion:**

Systematic analysis revealed structural diversity among tomato ZHD proteins, which indicates the possibility for diverse roles of *SlZHD* genes in different developmental stages as well as in response to abiotic stresses. Our expression analysis of *SlZHD* genes in various tissues/organs and under various abiotic stress and phytohormone treatments sheds light on their functional divergence. Our findings represent a valuable resource for further analysis to explore the biological functions of tomato *ZHD* genes.

**Electronic supplementary material:**

The online version of this article (10.1186/s12864-017-4082-y) contains supplementary material, which is available to authorized users.

## Background

Various regulatory proteins systematically controlled the many developmental processes in plants; among them, transcription factors (TFs) are major regulatory proteins with sequence-specific DNA or nucleotide binding activity [1–4]. TFs control a range of biological processes in plants, such as growth, development, metabolism, cell cycle progression, and responses to environmental stimuli. For example NF-Y, MYB, AP2, TCP, WRKY, NAC, GRF, and SPL TFs play important role in stress tolerances, whereas NAC, SPL, and GRF TFs are involved in root growth, flower, seed development, and plant transition [5]. Zinc finger homeodomain (ZHD) TFs, harboring a homeodomain (HD) and a C2H2-type zinc finger motif (ZF), are involved in plant development and stress responses [3]. ZHD TFs have distinct sequence characteristics compared to other plant HD-containing proteins identified to date [3, 6].

The HD is a DNA-binding domain containing approximately 60 amino acids that is present in numerous transcription factors in all eukaryotic organisms [7, 8]. HD proteins participate widely in development by regulating the expression patterns of target genes in both plants and animals [7]. Most HD proteins are associated with additional

domain(s) or motifs for protein–protein interactions and/or other regulatory functions [9]. HD-containing proteins are classified into six distinct families based on the presence of different motifs: leucine zipper-associated HD (HD-Zip), zinc finger motif-associated HD (ZF-HD), WUSCHEL-related homeobox (WOX), Bell type HD, finger domain associated to a HD (PHD finger), and Knotted-related homeobox (KNOX) proteins [10].

The zinc finger, one of the most important structural motifs, consists of a zinc ion in the core surrounded by several amino acid residues (cysteines or histidines in most cases) [11]. Zinc finger domains are widely present in many regulatory proteins and are actively involved in sequence-specific binding to DNA/RNA and in protein–protein interaction [11–13]. Zinc finger motifs are classified into different categories based on the presence of Cys and His residues, for instance, C3H, C2H2, and C2C2 [11]. Zinc finger TFs, especially C2H2-type TFs, play crucial roles in many metabolic pathways in plants, including stress responses and defense activation [14].

A ZHD protein was identified in *Flaveria trinervia* as a potential regulator of the gene encoding C4 phosphoenolpyruvate carboxylase (PEPCase) [15]. ZHD protein family members were subsequently identified in several model plants including *Arabidopsis thaliana* with 17 members and rice (*Oryza sativa*), with 15 members [8, 15]. In Arabidopsis, ZHD proteins act as transcriptional regulators with unique biochemical properties that function in the regulation of floral development [16]. *AtZHD1* is a transcriptional regulator that binds to the promoter region of *ERD1* (*EARLY RESPONSE TO DEHYDRATION STRESS 1*), and its expression is induced by drought, salinity, and abscisic acid (ABA) [17]. The overexpression of *NAC* and *AtZHD1* increases drought tolerance in Arabidopsis [20]. Soybean *ZHD1* and *2* (*GmZF-HD1* and *GmZF-HD2*) are upregulated upon pathogen inoculation, and GmZF-HD1 and GmZF-HD2 bind to the promoter region of a gene encoding calmodulin isoform 4 (*GmCaM4*) [18]. Three Arabidopsis MINI ZINC FINGER (AtMIF) proteins and their homologs share high levels of sequence similarity with the ZF domain of Arabidopsis ZHD proteins [19]. The presence of only a zinc finger motif without a HD in *MIF* genes suggests that MIF proteins might interfere with the functions of ZHD proteins via their ZF domains [8, 19]. Phylogenetic and sequence analyses of *ZHD* and *MIF* genes demonstrated that both are land plant-specific and that ZHDs and MIFs belong to two different groups of the ZHD protein family [8]. However, the origin, evolutionary history, and relationship of those two groups remain unclear [8].

As ZHD protein family members function as transcriptional regulators of floral development and stress responses in Arabidopsis, it is possible that they play similar roles in tomato (*Solanum lycopersicum*). Although *ZHD* genes have been investigated in Arabidopsis and several other species, no systematic, comprehensive investigation of the ZHD subfamily has been reported for any solanaceous crop. Therefore, in this study, we performed comprehensive genome-wide analysis of the *ZHD* gene family in tomato to explore their potential roles in organ development and responses to a wide range of stresses. We also analyzed the predicted gene structures, chromosomal locations, duplication events, and evolutionary divergence of the tomato *ZHD* genes and classified them based on phylogenetic analysis. Finally, we predicted the functions of *ZHD* genes based on their expression profiles and the presence of putative cis-elements in their upstream promoter regions. Our results lay the foundation for further studies aimed at uncovering the important biological functions of ZHD proteins in plants.

## Results

### Identification of *ZHD* family genes in tomato

We identified a total of 22 non-redundant putative *ZHD* genes, which we designated *SlZHD1*–*SlZHD22* (Sl for *Solanum lycopersicum*, Z for zinc finger, HD for homeodomain) according to their physical locations on the chromosomes (Table 1). The lengths of the open reading frame (ORF) of tomato *ZHD* genes range from 252 bp (*SlZHD14*) to 2418 bp (*SlZHD22*). The deduced encoded proteins of tomato ZHDs range in size from 83 aa (SlZHD14) to 805 aa (SlZHD22). In addition, their isoelectric points (pIs) range from 5.76 (SlZHD12) to 9.75 (SlZHD21) and their molecular weights (MWs) range from 9037.14 kDa (SlZHD14) to 90,496.49 kDa (SlZHD22). Information about these genes, including the chromosome locations and introns, is provided in Table 1.

**Table 1:** Detailed information about the *SlZHD* genes and corresponding proteins in tomato

Gene name	Locus name	ORF (bp)	Location	Length (aa)	Domain (start-end)	Mol. wt (kDa)	pI	Exons	Subcellular localization
*SlZHD1*	Solyc01g014970	690	SL2.50ch01:16,338,200…16,339,565 (−strand)	229	20–71	25,789.04	8.12	2	Extracellular
*SlZHD2*	Solyc01g102980	873	SL2.50ch01:91,639,500…91,641,389 (+ strand)	290	64–118	32,600.98	7.76	2	Nuclear
*SlZHD3*	Solyc01g103810	351	SL2.50ch01:92,343,498…92,343,848 (− strand)	116	14–68	13,144.06	9.46	1	Cytoplasmic
*SlZHD4*	Solyc01g103820	285	SL2.50ch01:92,348,106…92,348,390 (− strand)	94	14–68	10,361.66	8.27	1	Cytoplasmic
*SlZHD5*	Solyc01g103830	633	SL2.50ch01:92,352,642…92,353,696 (− strand)	210	10–64	24,492.28	9.68	2	Extracellular
*SlZHD6*	Solyc01g103840	255	SL2.50ch01:92,360,326…92,360,580 (− strand)	84	10–64	9529.72	6.27	1	Extracellular
*SlZHD7*	Solyc02g067310	879	SL2.50ch02:37,494,131…37,495,541 (+ strand)	292	62–118	32,086.75	8.20	2	Nuclear
*SlZHD8*	Solyc02g067320	996	SL2.50ch02:37,520,623…37,521,618 (− strand)	331	53–109	36,516.54	7.75	1	Nuclear
*SlZHD9*	Solyc02g067330	309	SL2.50ch02:37,527,458…37,527,766 (− strand)	102	29–83	11,208.36	6.18	1	Cytoplasmic
*SlZHD10*	Solyc02g085160	882	SL2.50ch02:48,141,575…48,142,456 (− strand)	293	89–143	33,312.95	8.14	1	Nuclear
*SlZHD11*	Solyc02g087970	273	SL2.50ch02:50,205,827…50,206,099 (+ strand)	90	23–75	10,104.24	9.07	1	Cytoplasmic
*SlZHD12*	Solyc03g061620	267	SL2.50ch03:31,305,510…31,305,776 (− strand)	88	23–77	9648.53	5.76	1	Cytoplasmic
*SlZHD13*	Solyc03g098060	540	SL2.50ch03:60,406,311…60,406,850 (− strand)	179	7–64	19,951.89	8.47	1	Nuclear
*SlZHD14*	Solyc03g116070	252	SL2.50ch03:65,585,458…65,585,709 (− strand)	83	21–74	9037.14	8.75	1	Cytoplasmic
*SlZHD15*	Solyc04g014260	744	SL2.50ch04:4,560,854...4561970 (− strand)	247	54–107	27,722.19	7.16	2	Nuclear
*SlZHD16*	Solyc04g074990	432	SL2.50ch04:60,885,156...60886298 (+ strand)	143	45–99	16,096.89	5.97	3	Cytoplasmic
*SlZHD17*	Solyc04g080490	873	SL2.50ch04:64,650,886…64,652,793 (− strand)	290	57–111	31,547.28	8.72	2	Nuclear
*SlZHD18*	Solyc05g007580	894	SL2.50ch05:2,120,151…2,121,044 (− strand)	297	52–108	32,814.23	7.26	1	Nuclear
*SlZHD19*	Solyc05g018740	360	SL2.50ch05:23,129,391...23129750 (− strand)	119	14–68	13,674.54	9.30	1	Cytoplasmic
*SlZHD20*	Solyc05g020000	360	SL2.50ch05:25,503,046...25503405 (− strand)	119	14–68	13,702.57	9.44	1	Cytoplasmic
*SlZHD21*	Solyc05g051420	504	SL2.50ch05:61,721,814...61724132 (− strand)	167	1–50	19,300.40	9.75	2	Nuclear
*SlZHD22*	Solyc09g089550	2418	SL2.50ch09:69,252,813...69257262 (− strand)	805	554–608	90,496.49	6.97	4	Extracellular

*ORF* Open reading frame, *bp* base pair, *aa* amino acid, *pI* isoelectric point, *kDa* kilodaltons

### Phylogenetic and gene structure analysis of the tomato *ZHD* gene family

**Fig. 1:**
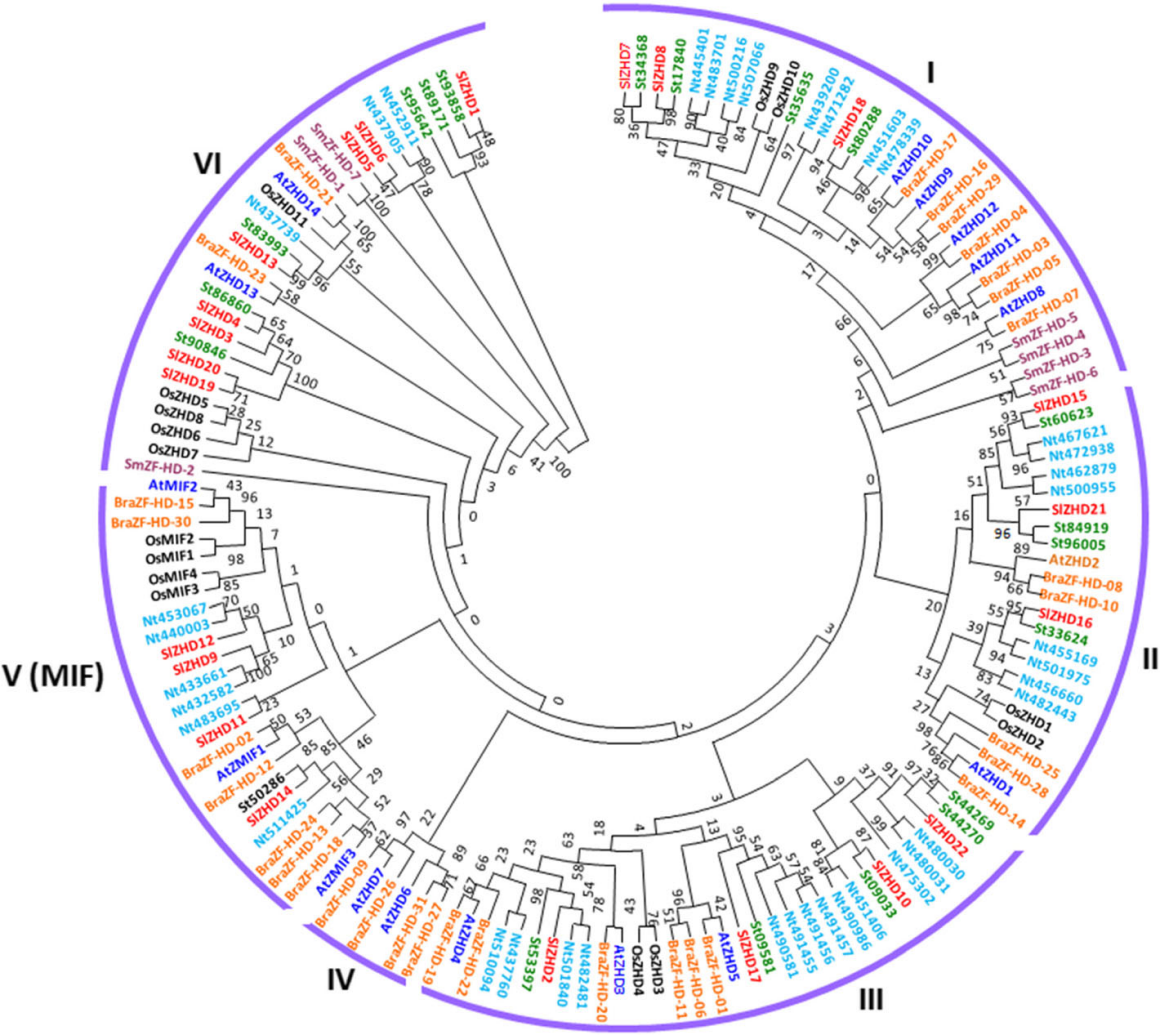
Phylogenetic relationship of Arabidopsis(AtZHD), rice(OsZHD), potato (St, *Solanum tuberosum* is used instead of PGSC0003DMT4000), tobacco (Nt, *Nicotiana tabacum* is used instead of XP_0164), Chinese cabbage (BraZF-HD), *Selaginella moellendorffii*, (SmZF-HD) and tomato (SlZHD) *ZHD* genes. The conserved ZF-HD_ dimer domain sequences of Arabidopsis, rice, potato, tobacco, Chinese cabbage, *Selaginella moellendorffii*, and tomato genes were aligned using ClustalX, and the tree were constructed by the neighbor-joining (NJ) method with MEGA 6.0. The numbers on the branches indicate bootstrap support values from 1000 replications. The protein sequences used in the phylogenetic analysis are listed in Additional file 4, along with their accession numbers. The tree was divided into six subfamilies according to bootstrap support values and evolutionary distances

To obtain insights into the evolutionary relationships among tomato ZHD family proteins, a phylogenetic tree was constructed from 150 amino acid sequences of tomato (22), potato (38), tobacco (20), Arabidopsis (17), rice (15), Chinese cabbage (31) and *Selaginella moellendorffii* (7) (Fig. 1). The ZHD protein family was divided into six well-conserved clades (I–VI), with significant bootstrap support [8, 20]. Among these, clade V contained the previously described MIF proteins from Arabidopsis, rice and Chinese cabbage together with four tomato ZHD proteins. Therefore, our analysis separated the MIF proteins from the other ZHD proteins. Clade IV contained the fewest ZHD members, with no SlZHD protein found in this clade. The largest number of SlZHD proteins was in Clade VI.

**Fig. 2:**
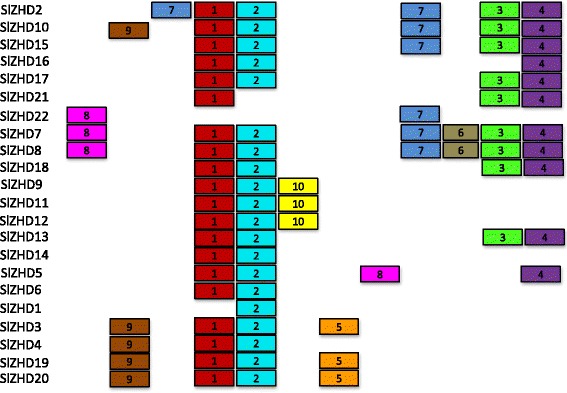
Schematic representation of the 10 conserved motifs in SlZHD proteins. SlZHD protein motifs were identified using the online MEME program. Members of same group are arranged sequentially according to phylogenetic classification. Different colored boxes represent different motifs, where the number in center of each boxes indicates their name (Motif 1 to 10). The colored boxes were drawn and ordered manually according to the results of MEME analysis. The length of each box in the figure does not represent the actual motif size in the proteins

To further investigate the diversity of the tomato *ZHD* genes, we analyzed SlZHD protein motifs using the MEME online server. Ten conserved motifs were identified, i.e., motif 1 to 10 (Fig. 2, Additional file 1: Fig. S1). An overview of these protein motifs is presented in Additional file 1: Fig. S1. Motif 1 and 2, the most common motifs, comprise the ZF-HD dimer domain. Among the 22 gene products, motif 1 was absent in SlZHD1 and motif 2 was absent in SlZHD21. Motif 10 was mainly found in subfamily III proteins (Figs. 1 and 2). Motif 9 was mainly present in subfamily VI, except for SlZHD10, a subfamily IV protein (Figs. 1 and 2). Motif 5 was mainly found in subfamily I and II proteins (Figs. 1 and 2). The subfamily-specific distribution of conserved motifs may have contributed to the functional divergence of *ZHD* genes in tomato.

**Fig. 3:**
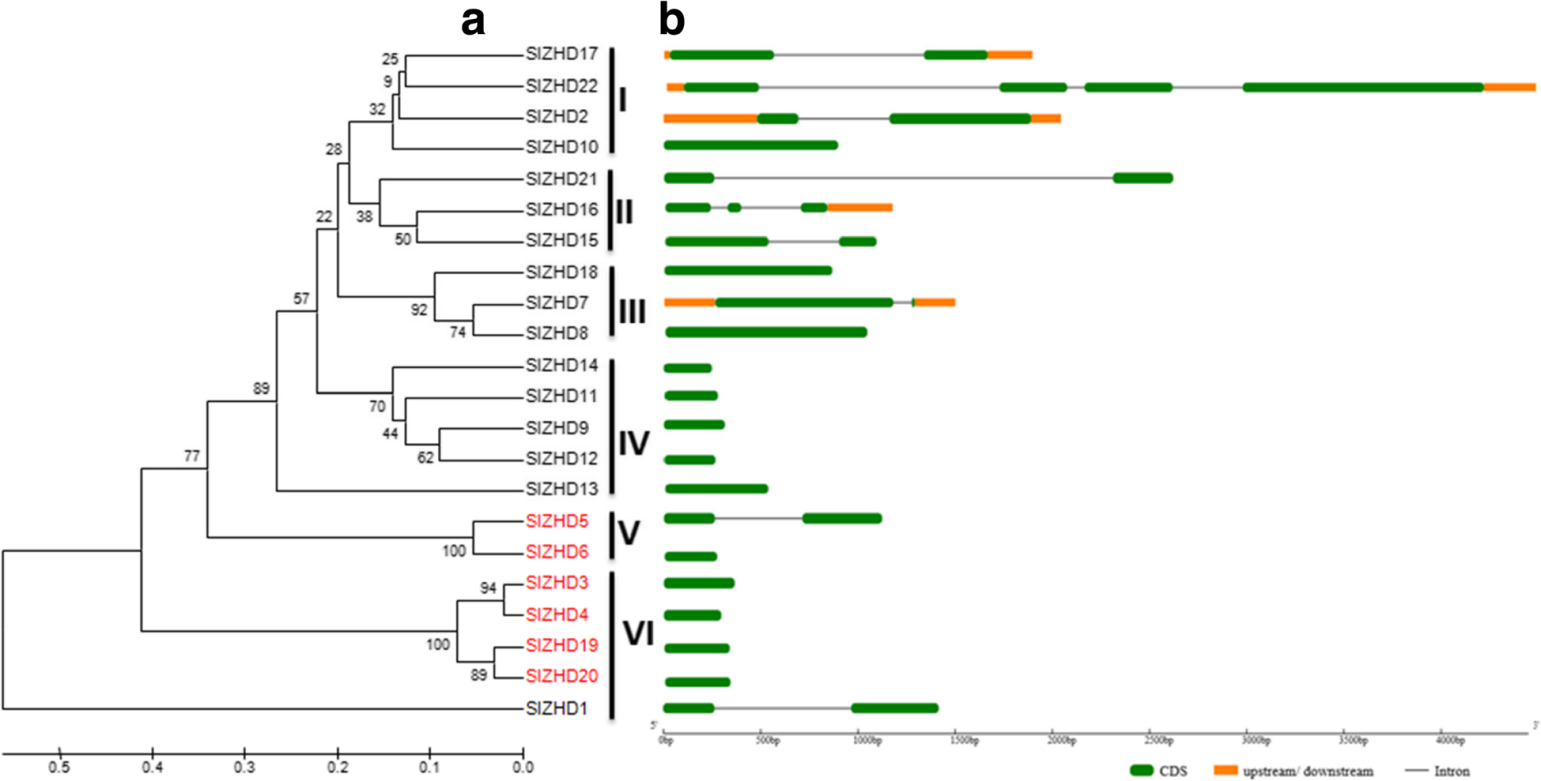
Phylogenetic relationships and gene structures of *SlZHD* genes. **a**. Phylogenetic tree constructed among the 22 *SlZHD* genes using full-length amino acid sequences with MEGA 6.0 following the UPGMA method with 1000 bootstrap replicates. **b**. Exon-intron organization of *SlZHD* genes. Exon and introns are represented by green boxes and gray lines, respectively. Untranslated regions are indicated by orange boxes

To gain further insights into the structural diversity of *ZHD* genes in tomato, we constructed a phylogenetic tree based on the 22 SlZHD proteins using their full-length protein sequences (Fig. 3a). The SlZHD proteins were also classified into six subfamilies in this phylogenetic tree, which is in an agreement with the results shown in Fig. 1 based on phylogenetic analysis of the seven plant species (Figs. 1 and 3a). Analyzing the genetic structural diversity among the proteins of a multigene family is a useful way to perform evolutionary analysis. We therefore deduced the exon-intron organization of individual *SlZHD* genes to examine their structural diversity (Fig. 3b). Most *SlZHD* genes (13 out of 22) lack introns, whereas the remaining nine have one to three introns. Most closely related members in the same subfamily share almost identical exon-intron organization (Fig. 3a and b). For example, *SlZHD* genes in subfamily I lack introns. However, the exon-intron organization was not always conserved for most sister gene pairs. For example, *SlZHD5/−6* and *SlZHD7/−8* have different numbers of exons and introns (Fig. 3a and b).

### Chromosomal location, gene duplication, and microsynteny analysis

**Fig. 4:**
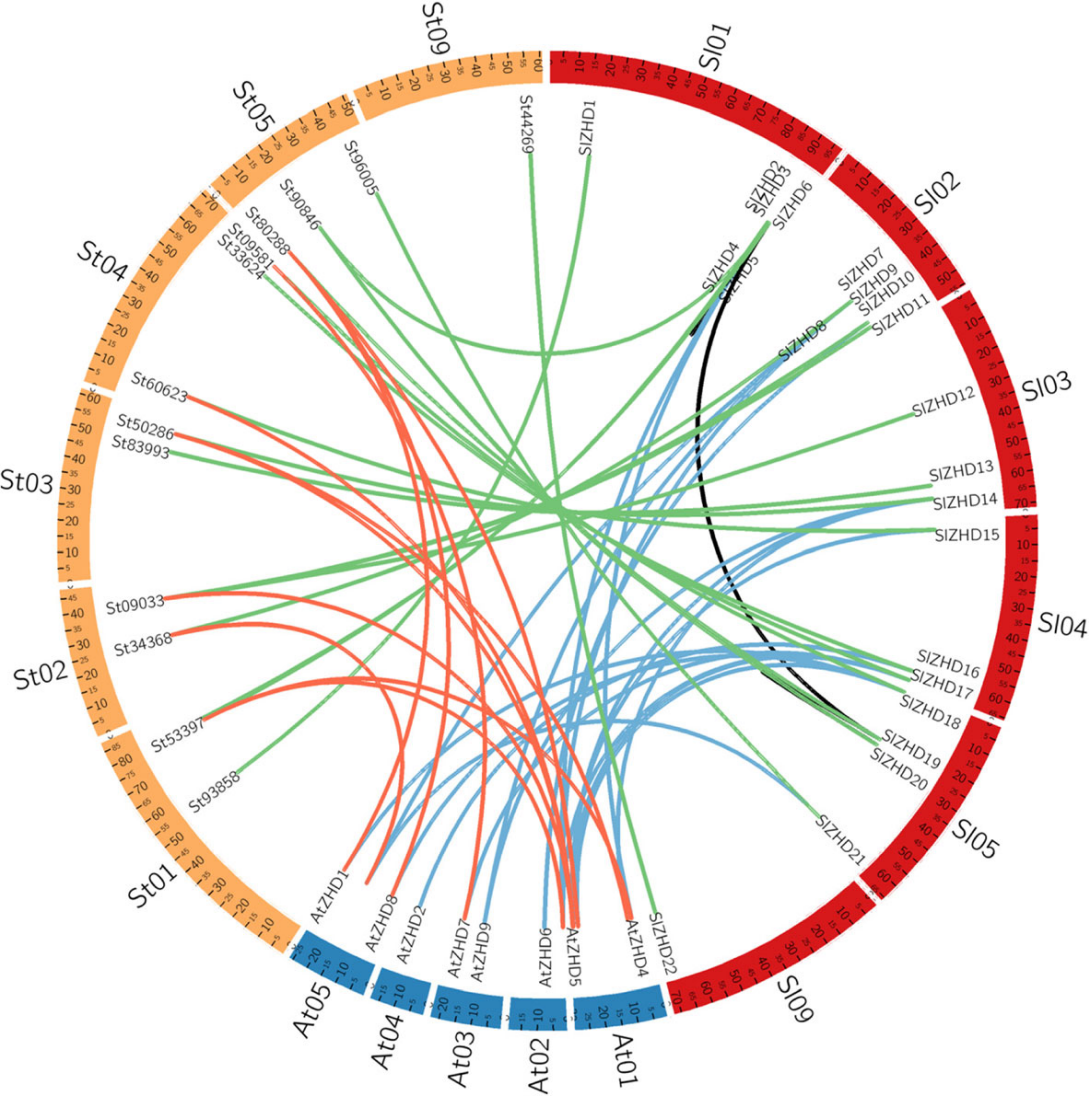
Microsynteny analyses of *ZHD* genes among *S. lycopersicum*, *S. tuberosum*, and *A. thaliana*. The chromosomes from the three species are indicated in different colors: red, yellow, and blue represent the *S. lycopersicum*, *S. tuberosum*, and *A. thaliana* chromosomes, respectively. Black lines represent duplicated *SlZHD* genes on tomato chromosomes

We mapped the 22 *SlZHD* genes onto the 12 tomato chromosomes (Additional file 1: Fig. S2), finding that they are unevenly distributed on six of the 12 chromosomes. Chromosome 1 contains the highest number of *ZHD* family genes (six genes), followed by chromosome 2 and 5 (five and four genes, respectively). Both chromosome 3 and 4 contain three genes, while chromosome 9 possesses only one gene, and no *ZHD* genes are present on the six remaining tomato chromosomes. To determine the segmental duplication events between the genes, we used the criteria [21]; when the query coverage percentage and identity of the candidate genes was ≥80% they were considered to be duplicated genes. Therefore, segmental duplication analysis showed that four pairs of *SlZHD* genes, *SlZHD3-SlZHD4*, *SlZHD5-SlZHD6*, *SlZHD19-SlZHD20*, and *SlZHD4-SlZHD19*, originated through segmental duplication (Additional file 2: Table S2). According to the criterion of tandem duplication (when two genes were separated by five or fewer genes within a 100-kb region on a chromosome), no pair of *SlZHD* genes originated by tandem duplication (Additional file 1: Fig. S2). To determine the selection constraints on the duplicated *SlZHD* genes, we estimated the Ka/Ks ratio of each pair of paralogous genes using the method of Nei & Gojobori [22] and found that the ratios for seven paralogous pairs <1 (Additional file 2: Table S2). This result suggests that these genes experienced strong purifying/negative selection pressure, with little variation taking place after duplication. The duplication of paralogous gene pairs is estimated to have occurred 4.3 to 10.13 million years ago (Mya) (Additional file 2: Table S2). Based on a comparative microsyntenic map of Arabidopsis versus tomato and potato (*S. tuberosum*), 15 pairs of Z*HD* orthologous genes between *S. lycopersicum* and *S. tuberosum*, 16 pairs between *A. thaliana* and *S. lycopersicum*, whereas, 11 pairs of orthologous gene pairs were found between *A. thaliana* and *S. tuberosum* (Fig. 4). These results confer that during species divergence 16 tomato *ZHD* and 11 potato *ZHD* genes are derived from *Arabidopsis*.

### Expression profiling of tomato *ZHD* genes in various organs

**Fig. 5:**
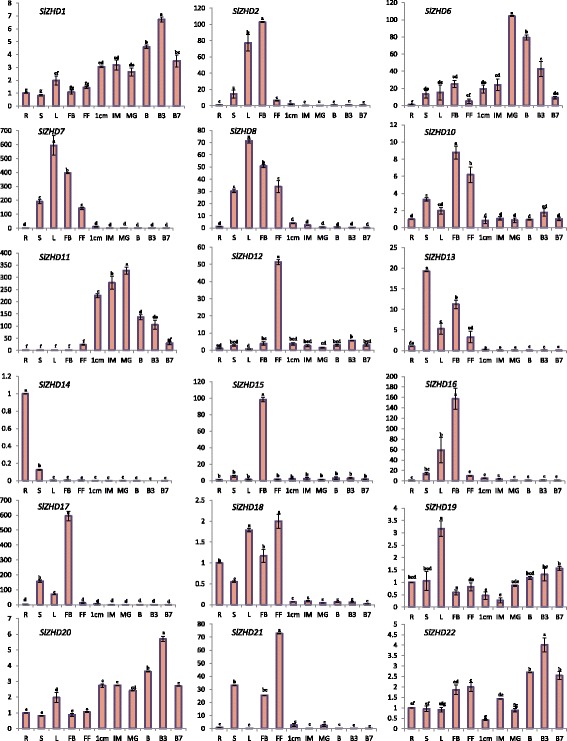
Expression profiles of *SlZHD* genes in various tomato tissues. Root (R), stem (St), meristem (M), leaves (L), flower bud (FB), full blooming flower (FF), and fruits at six developmental stages (1 cm: 1 cm-sized fruit, IM: immature fruit, MG: mature green fruit, B: breaker, B3: 3 days after breaker, B7: 7 days after breaker) analyzed by qRT-PCR. Relative gene expression levels were normalized to *EF1a* expression levels. Error bars represent standard deviations of the means of three independent replicates. Statistically significant variations in expression and mean values at different sampling points (ANOVA, *p < 0.01* for all 12 genes) are indicated with different letters. Y axis indicates the relative expressions of the genes

To obtain a first glance at the roles of *SlZHD* genes during various developmental process of tomato, the transcript accumulation levels were investigated in 13 tomato organs (using root, stem, leave, flower bud, full blooming flower, and six fruit developmental stages) by qRT-PCR. Most *ZHD* genes, except for *SlZHD3*, *SlZHD4*, *SlZHD5*, and *SlZHD9*, exhibited tissue-specific expression patterns (Fig. 5). Four genes (*SlZHD3*, *SlZHD4*, *SlZHD5*, and *SlZHD9*) were not expressed in any of the organs examined. These genes exhibited little or no expression in the RNA-Seq data set (Additional file 3). Alternatively, we may have failed to detect their expression in the organs/under the conditions examined, or these four genes might be expressed in other organs or could be pseudogenes. Five genes (*SlZHD2*, *SlZHD10*, *SlZHD15*, *SlZHD16*, and *SlZHD17*) and two genes (*SlZHD12* and *SlZHD21*) were highly expressed in flower buds and fully open flowers, respectively, compared to vegetative tissue and developing fruits (Fig. 5). By contrast, three genes (*SlZHD7*, *SlZHD8*, and *SlZHD19*) were strongly expressed in leaves, one gene (*SlZHD13*) was highly expressed in stems, and one gene (*SlZHD14*) was highly expressed in roots and stems compared to reproductive tissue and developing fruits. In addition, eight genes (*SlZHD1*, *SlZHD6*, *SlZHD10*, *SlZHD11*, *SlZHD12*, *SlZHD19*, *SlZHD20*, and *SlZHD22*) exhibited differential expression profiles in fruits at six developmental stages (Fig. 5). Among these, three genes (*SlZHD1*, *SlZHD20*, and *SlZHD22*) were highly expressed at B3 (breaker stage [after 3 days]), two (*SlZHD6* and *SlZHD11*) were highly expressed at MG (mature green stage), and one (*SlZHD19*) was highly expressed at the ripening stages (B, B3, and B7) of fruit development (Fig. 5). Similar expression patterns were found in different organs for some paralogous gene pairs, e.g., *SlZHD7* and *SlZHD8* were strongly expressed in leaves, followed by flower buds (Figs. 3 and 5). However, some gene pairs exhibited differential expression patterns in different organs. For example, *SlZHD16* was highly expressed in flower buds, whereas its paralog, *SlZHD21*, was highly expressed in flowers (Figs. 3a and 5).

### Expression profiling of tomato *ZHD* genes in response to abiotic stress and phytohormone treatment

**Fig. 6:**
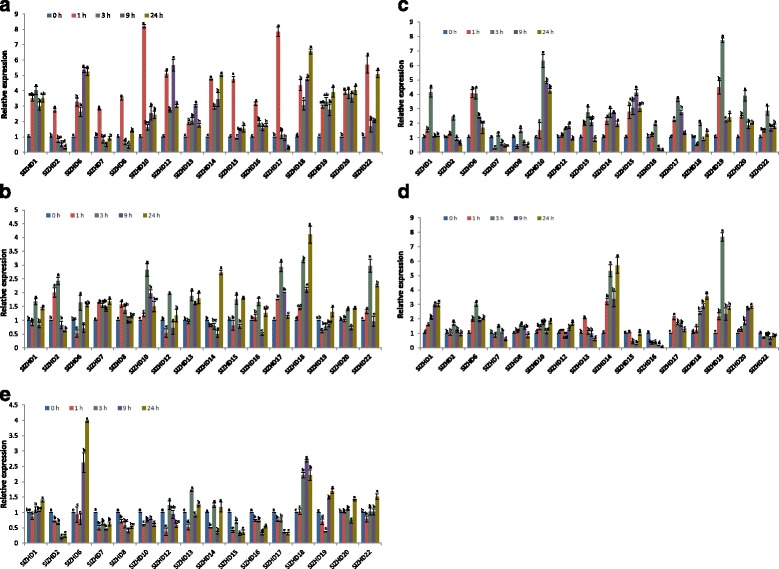
Expression analysis of 16 *SlZHD* genes by qRT-PCR: the relative expression levels of *SlZHD* genes under different abiotic and phytohormone treatments: **a** drought, **b** NaCl, **c** heat, **d** cold, **e** ABA; error bars indicate the standard error among three replicates. Different letters associated with each treatment indicate statistically significant differences at the 5% level, where the same letter indicates that the values did not differ significantly at *P* < 0.05 according to Tukey’s pairwise comparison tests

We analyzed the responses of the *SlZHD* genes to abiotic stress (drought, NaCl, heat, and cold) and phytohormone (ABA) treatment (Fig. 6). The expression of *SlZHD11* and *SlZHD21* did not differ between control and abiotic stress- or phytohormone-treated plants. However, many genes were up- or downregulated by these treatments at various time points.

Drought treatment caused a marked change in the transcription levels of 16 *SlZHD* genes at different time points (Fig. 6a). Eleven of the 16 genes (except *SlZHD2*, *SlZHD7*, *SlZHD8*, *SlZHD15*, and *SlZHD17)* were significantly upregulated (2- to 8-fold) within 24 h after treatment compared to the control. More importantly, the expression levels of most of these genes peaked during early stages of treatment. For instance, *SlZHD2*, *SlZHD7*, *SlZHD8*, *SlZHD10*, *SlZHD15*, *SlZHD16*, and *SlZHD17* exhibited maximum expression (more than 2.5- to 8-fold vs. the control) at 1 h after treatment. *SlZHD12* and *SlZHD14* were the most highly expressed at 1 h and at 9 h after treatment, and *SlZHD6* expressed peaked at 9 h and 24 h after treatment. The expression of *SlZHD22* peaked at both early and last time points (1 h and 24 h after treatment). By contrast, the expression of *SlZHD18* and *SlZHD19* peaked at 24 h after drought treatment.

The expression levels of 16 *SlZHD* genes changed in response to NaCl treatment (Fig. 6b). Seven of the 16 *SlZHD* genes (*SlZHD1*, *SlZHD6*, *SlZHD12*, *SlZHD15*, *SlZHD16*, *SlZHD20*, and *SlZHD22*) exhibited maximum expression (more than 1.5- to 4-fold vs. the control) at 3 h and 24 h of treatment, whereas two genes (*SlZHD14* and *SlZHD19*) were the most highly upregulated only at last time point (24 h) compared to the control. *SlZHD7* and *SlZHD8* were slightly upregulated (0.5- to 1.5-fold) from 1 to 24 h, whereas *SlZHD18* expression peaked at 24 h (relative expression ~4-fold higher) compared to the control. *SlZHD10* and *SlZHD13* were upregulated (1- to 2-fold) from 3 h to 24 h of treatment compared to the control. *SlZHD2* was strongly induced at 1 h and 3 h, where *SlZHD17* was significantly upregulated at 1 h to 9 h after treatment as compared to the control.

Under heat treatment, *SlZHD6*, *SlZHD14*, *SlZHD15*, *SlZHD19*, *SlZHD20*, and *SlZHD22* were significantly upregulated at various time points compared to the control (Fig. 6c). The expression of *SlZHD7*, *SlZHD8*, and *SlZHD12* was relatively low (<0.5-fold control levels) under heat treatment, whereas *SlZHD10* was strongly upregulated (>4- to 6.5-fold) at 1 h to 24 h after treatment. *SlZHD13* and *SlZHD17* genes were upregulated by >1- to 3-fold from 3 h to 9 h of heat treatment compared to the control. Four genes (*SlZHD1*, *SlZHD2*, *SlZHD16*, and *SlZHD18*) were also expressed at higher levels (>1- to 3.5-fold) at 1 h of treatment compared to the control.

The expression of *SlZHD2*, *SlZHD7*, *SlZHD8*, *SlZHD10*, *SlZHD12*, *SlZHD15*, *SlZHD16*, and *SlZHD22* was not significantly altered (<0.5-fold) by cold treatment (Fig. 6d). By contrast, *SlZHD1*, *SlZHD18*, and *SlZHD20* were upregulated by this treatment, whereas *SlZHD16* was downregulated from 1 h to 24 h after treatment vs. the control. *SlZHD6*, *SlZHD14*, and *SlZHD19* were significantly upregulated (>1- to 7-fold) from 1 h to 24 h after cold treatment compared to the control, and *SlZHD13* was induced at 1 h after treatment compared to the control.

Under ABA treatment, seven of the 16 genes (*SlZHD2*, *SlZHD7*, *SlZHD8*, *SlZHD10*, *SlZHD15*, *SlZHD16*, and *SlZHD17*) were downregulated compared to the control (Fig. 6e). *SlZHD6* and *SlZHD19* were significantly induced at 9 and 24 h after ABA treatment compared to the control, and three genes (*SlZHD1*, *SlZHD20*, and *SlZHD22*) were slightly upregulated at 24 h after treatment compared to the control. *SlZHD18* was highly expressed (> 2.5- to 3-fold) at 3 h to 24 h after treatment compared to the control, while *SlZHD12*, *SlZHD13*, and *SlZHD14* exhibited constitutively weak expression levels under ABA treatment. The expression of *SlZHD12* and *SlZHD13* declined at 1 h, but these genes were slightly upregulated at the remaining time points compared to the control. Finally, *SlZHD14* was upregulated at 3 and 24 h after treatment compared to the control.

### Putative stress- and hormone-responsive cis-elements in the promoter regions of *SlZHD* genes

Cis-acting elements in the promoter region of genes have vital roles in determining the tissue-specific or stress-responsive expression patterns of genes under variable environmental conditions [23]. Significant positive correlations have been reported between the density of cis-elements and multi-stimulus response genes in upstream regions [24]. A web search was performed using PlantCare database to identify possible stress and hormone-responsive cis-acting elements in the promoter regions of tomato *SlZHD* genes. Cis-elements responsive to developmental cue, tissue specific expression, and stress responses are found in the promoter of *SlZHD* family members. In addition, we found five abiotic-stress responsive cis-elements: MBS (MYB binding site) present in 11 different *SlZHD* genes responsive to drought, ABRE (ABA-responsive element) in 11 genes, HSE (heat stress responsiveness) in 12 genes, LTR (low-temperature responsiveness) in 3 genes, and DRE (dehydration, low-temp, salt stresses) in one *SlZHD* gene (Additional file 2: Table S3). Among those genes, we found higher expression of *SlZHD10*, *SlZHD12*, *SlZHD18* and, *SlZHD22*, which also bear the cis-element MBS for drought stress responsiveness. In addition, ABA-induced upregulation was found in case of *SlZHD18* and *SlZHD22*, coinciding with the presence of ABRE cis-element. The LTR is found in *SlZHD1*, in agreement with the cold-induced higher expression of that gene. The heat responsive cis-element HSE was found in the genes *SlZHD1*, *SlZHD6*, *SlZHD10*, *SlZHD19*, *SlZHD20*, and *SlZHD22*, consistent with their heat stress-induced upregulation (Fig. 6a-e and Additional file 2: Table S3).

## Discussion

The plant-specific *ZHD* gene family has been found in major groups of land plants, but not in algae [8], suggesting that these genes may have evolved before the divergence of land plants but after the separation of land plant lineages from single-celled algae. In the current study, we identified and characterized 22 *ZHD* genes from tomato via genome-wide analyses. The number of *ZHD* genes in tomato was somewhat higher than that of Arabidopsis (17), rice (15), and *Selaginella moellendorffii* (7) but lower than that of *Brassica rapa* (31). However, compared with the differences in overall genome size between tomato (950 Mb) and smaller-genomes plants such as Arabidopsis (164 Mb), rice (441 Mb), *B. rapa* (283.8 Mb), and *Selaginella moellendorffii* (212.5 Mb), the number of *ZHD* genes in tomato was relatively small, suggesting that genome duplication events might have contributed to the expansion of the *ZHD* gene families in plants with smaller genome sizes*.* For instance, four different large-scale duplication events occurred in the Arabidopsis genome, and more than half of the *AtZHD* genes likely arose as a result of genome duplication [8, 25, 26]. The deduced protein parameters and conserved ZF-HD_dimer domains of *SlZHD* family genes are consistent with those of other plant species [3, 8], indicating that ZHD proteins are structurally similar. The 22 SlZHD proteins were classified into six subfamilies. Among these, two subfamilies (I and VI) including proteins found in seedless vascular plants, eudicots, and/or monocots, suggesting that these proteins might have been generated during the early evolution of land plants, considerably before the divergence of major groups of angiosperms (Fig. 1). MIF proteins from various crops form a phylogenetically distinct clade from ZHD proteins, suggesting structural divergence among these proteins and that *MIF* genes might be derived from *ZHD* genes after losing the HD [8]. Analysis using the MEME server identified various conserved motifs in SlZHD proteins, with similar motifs found in the most closely related members in the phylogenetic tree, revealing the functional similarity among the same subfamily proteins (Figs. 1 and 2). Gene structure analysis confirmed that 13 of the 22 *SlZHD* genes lack introns, whereas the nine remaining genes contain one to three introns. The majority of plant *ZHD* genes were previously found to be intronless [3, 8, 20], but our data do not support this finding for tomato. Three main mechanisms, including exon/intron gain/loss, exonization/pseudo-exonization, and insertion/deletion, are responsible for the variation in exon-intron structures of a gene, with each mechanism contributing to the structural divergence of genes alone or in combination [27, 28]. The variable exon-intron structures of *ZHD* genes observed in tomato compared to other plants suggests that there is structural divergence in this gene family in *S. lycopersicum*. Moreover, the similar exon-intron organization in different subfamilies suggests that these genes were highly conserved during evolution (Fig. 3).

Gene duplication mechanisms, including segmental duplication, tandem duplication, and transposition (retro and replicate transposition), are important contributors to biological evolution [29]. Among these, segmental duplication is a principal contributor to the amplification of many gene families [30]. In the current study, we found that four pairs of paralogous genes developed through segmental duplication, whereas no evidence of tandem duplication was detected for any gene pair, indicating that segmental duplication rather than tandem duplication has played a prominent role in the expansion of the tomato *ZHD* gene family. The Ka/Ks ratios of the duplicated gene pairs indicate that they have undergone purifying selection during the process of evolution. Furthermore, our calculation of the duplication times of the paralogous gene pairs predicted that the segmental duplication event in the *SlZHD* gene family was occurred 4.3 to 10.13 Mya ago (Additional file 2: Table S2).

The expression of four *SlZHD* genes (*SlZHD3*, *SlZHD4*, *SlZHD5*, and *SlZHD9*) was not detected in any tissue examined, indicating that they might be pseudogenes or might be expressed only at specific developmental stages or under specific conditions not included in our study. ZHD transcription factors are involved in regulating various biological processes in plants, including development and responses to abiotic stress and phytohormones [3, 8, 20]. Our expression analysis indicates that most *SlZHD* genes have tissue-preferential expression patterns. In fact, five of the *SlZHD* genes were predominately expressed in flower buds, suggesting they play important roles in flower bud development (Fig. 5). Three *SlZHD* genes (*SlZHD7*, *SlZHD8*, and *SlZHD19*) were highly expressed in leaves, and two genes (*SlZHD12* and *SlZHD21*) were highly expressed only in flowers, suggesting they are involved in leaf and flower development. *ZHD* gene family members in other plants (e.g. Arabidopsis) are preferentially expressed in floral tissues, revealing their vital regulatory role in floral tissue development [6]. The root is the main organ responsible for water and nutrient acquisition in plants. The predominant expression of *SlZHD14* in roots suggests that it might be involved in root development and/or water and nutrient uptake.

Fruit development and subsequent growth is a multifaceted biological process that ultimately leads to the production of crops for harvest. Fruit development is controlled by various transcriptional regulatory networks involving transcription factors such as members of the NAC, MADS-box, and EIN3/EIL families [31]. However, to date, potential roles of ZHD family proteins in fruit development have been characterized only in grape (*Vitis vinifera*) [20]. In the current study, the transcript levels of five *SlZHD* genes (*SlZHD1*, *SlZHD19*, *SlZHD20*, and *SlZHD22*) were generally higher in ripening tomato fruit compared to other stages (B, B3), suggesting they might function in tomato fruit growth and development, especially at the ripening stage. *SlZHD11* was upregulated during the early and later phases of green fruit development (1 cm, IM and MG), indicating its potential involvement in cell division and elongation, perhaps to increase fruit size. *SlZHD6* expression was highest at the MG stage and then gradually declined thereafter, suggesting it might be involved in increasing fruit size and shape (Fig. 5). Similarly, tissue-specific expression was detected for *ZHD* family genes in Arabidopsis, Chinese cabbage, maize, and grape across a variety of tissues [3, 8, 20, 32]. The similar expression patterns of duplicated paralogous gene pairs (i.e., *SlZHD19* and *SlZHD20*) suggests that their functions might have been conserved even after duplication and that they might play redundant roles in regulating tissue development. However, the diverse expression patterns of several pairs of paralogs suggest that they likely play different roles in tomato development.

Transcription factors having specific DNA binding motifs (such as zinc fingers, MYB motif) are induced by various signals during specific developmental processes and under various stress conditions [33, 34]. ZHD family proteins contain a DNA binding motif and a zinc finger type motif, suggesting that when they are induced by various signals under different environmental conditions they could play an important role in mediating adaptive responses to various stresses. *ZHD* genes from Arabidopsis were recently shown to be induced by various abiotic stresses including drought and salt stress, as well as ABA treatment [35]. Therefore, we investigated the expression profiles of *ZHD* genes in tomato after stress treatment, finding that the expression patterns of many genes differed in response to stress treatments (Fig. 6). *SlZHD2*, *SlZHD7*, *SlZHD8*, and *SlZHD15* were upregulated within 1 h of drought and NaCl treatment, but their expression was not markedly altered by cold stress. *SlZHD18* was markedly induced by drought, NaCl, and cold treatment, but not by heat stress. Therefore, *SlZHD* genes might play important roles in various abiotic stress responses. Particularly, the transcript levels of two segmentally duplicated genes, *SlZHD19* and *SlZHD20*, markedly increased from 1 h to 24 h of drought, heat, and cold stress treatment, whereas we detected only minor changes in their expression in response to NaCl stress, supporting the involvement of these genes in the responses to drought, heat, and cold stress rather than salinity stress.

Transcription factors are involved in the regulation of stress signaling and stress-responsive gene expression through various mechanisms that rely on a combination of cis-acting elements present in numerous stress-related genes [2, 36]. Many stress-associated cis-acting elements have been identified in plants [37]. In the current study, several stress-responsive cis-elements were widely found in the promoter regions of most abiotic stress-induced *ZHD* genes in tomato (Additional file 2: Table S3). Overall 16 *SlZHD* genes were differentially regulated (up−/downregulated) in response to at least one stress condition, and the presence of stress responsive cis-elements suggests that these genes play important roles in regulating gene expression in response to abiotic stress and in environmental adaptation. Further studies on the putative cis-elements in tomato *ZHD* genes are needed to unravel the complex regulatory mechanisms involving the cis-elements and stress tolerance in tomato. *AtZHD1* is related to some *NAC (NAM/ATAF1/2/CUC2)* genes, and the overexpression of *AtZHD1* and *NAC* increases drought tolerance in Arabidopsis [38]. In addition, 31 Chinese cabbage *ZHD* genes were found to be regulated by abiotic and hormonal stress [3].

ABA is a key regulator in the adaptation of plants to unfavorable environmental conditions such as high salinity, drought, and cold [39]. Almost all 16 *SlZHD* genes were differentially regulated by ABA, with many (*SlZHD2*, *SlZHD7*, *SlZHD8*, *SlZHD10*, *SlZHD15*, *SlZHD16*, and *SlZHD17*) downregulated by this treatment. Two genes, *SlZHD6* and *SlZHD18*, were markedly upregulated from 9 h to 24 h and 3 h to 24 h after ABA treatment, respectively. The five remaining genes had different expression patterns at different time points, suggesting that *ZHD* genes play regulatory roles in stress responses by modulating ABA signaling. Indeed, *ZHD* genes are regulated by ABA stress in several plant species, including *AtZHD1* in Arabidopsis, *VvZHD4* and *VvZHD*13 in grape, and *TaZHD1* in wheat [33, 40–42].

Most *SlZHD* genes within the same subfamily in the phylogenetic tree had different expression patterns. For example, *SlZHD15* and *SlZHD16* in subfamily II showed diverse expression patterns in response to all abiotic stress treatment, indicating that the regulatory sequences in these genes that respond to stress conditions have diverged significantly during gene evolution. However, some genes in the same subfamily showed similar expression patterns under all treatments, such as *SlZHD7* and *SlZHD8*, indicating that the regulatory sequences in these genes that respond to stress conditions share high sequence similarity and were conserved during the course of evolution.

## Conclusions

*ZHD* genes have been comprehensively characterized in several model plant species. However, to the best of our knowledge, no systematic study of this gene family has been performed in any Solanaceous species. In this study, we identified 22 *ZHD* genes in *Solanum lycopersicum*. Our systematic analysis revealed some structural diversity among tomato ZHD proteins, suggesting they play diverse roles in plant adaptation to environmental stress during particular stages of development. Our expression profiling analysis of *SlZHD* genes in various tissues/organs and under different abiotic stress and phytohormone (ABA) treatments should facilitate the identification of appropriate candidate genes for further functional characterization. The information obtained in this study lays the foundation for further analysis of the biological functions of ZHD proteins in tomato.

## Methods

### Identification of *ZHD* genes and encoded proteins in tomato

To conduct genome-wide characterization of *ZHD* family gene (Z for Zinc finger, HD for homeodomain) in tomato, the keyword ZF-HD was used as a query identify *ZHD* genes in the tomato genome using the Sol Genomics Network (SGN) (https://www.solgenomics.net//) [43]. After removing redundant sequences using blast search of conserved domain in NCBI (https://blast.ncbi.nlm.nih.gov/Blast.cgi) 22 *ZHD* genes were identified and confirmed through comparisons with the iTAK (Plant Transcription factor and Protein Kinase Identifier and Classifier) database (http://bioinfo.bti.cornell.edu/cgi-bin/itak/index.cgi) [44] and the Tomato Genomic Resources Database (TGRD) (http://webcache.googleusercontent.com/search?q=cache:UgWJjBHR92EJ:59.163.192.91/tomato2/contact.html+&cd=2&hl=en&ct=clnk&gl=kr = zf-HD) [45]. All of the predicted ZHD proteins had typical “ZF-HD” dimer domains (Pfam accession number PF04770), which were confirmed by comparing these sequences with previously identified Arabidopsis ZHD domain sequences using the web tool from EMBL (http://smart.embl-heidelberg.de/) [46]. Protein sequences and CDS of the identified tomato *ZHD* genes were obtained from SGN (https://solgenomics.net/) [43]. TGRD (http://59.163.192.91/tomato2/getTF_family.php?trans_fac_family = zf-HD) [45] and iTAK (http://bioinfo.bti.cornell.edu/cgi-bin/itak/index.cgi) [44] were used to verify the identified sequences.

### Sequence analysis of tomato *ZHD* genes

The primary structures including the length, molecular weight (Mw), and isoelectric point (pI) of the deduced tomato ZHD proteins were analyzed using ProtParam (http://web.expasy.org/protparam/) [47] (Table 1). The tomato *ZHD* CDS and their corresponding genomic sequences were aligned with the GSDS (http://gsds.cbi.pku.edu.cn/) [48] web tool to analyze the exon/intron structures of the tomato *ZHD* genes. MEME software (http://meme-suite.org/) [49] was employed to analyze the tomato ZHD protein motifs. The distinctive motifs were identified using the following parameters: (1) width of optimum motif ≥6 and ≤500 nt; (2) maximum number of motifs 10. The similarity among the 22 tomato ZHD proteins was analyzed using the NCBI protein BLAST tool (https://blast.ncbi.nlm.nih.gov/Blast.cgi) [50]*.* The subcellular locations of the ZHD proteins in *S. lycopersicum* were predicted using ProtComp 9.0 from SoftBerry (http://linux1.softberry.com/berry.phtml) [51].

### Phylogenetic analysis of tomato ZHD proteins

The deduced amino acid sequences of Arabidopsis, rice, and Chinese cabbage ZHD proteins containing ZF-HD dimer domain were obtained from TAIR (https://www.arabidopsis.org/) [52], TIGR-Rice Genome Annotation Project (http://rice.plantbiology.msu.edu/) [53], and the BRAD *Brassica* database (http://brassicadb.org/brad/) [54], respectively. The amino acid sequences from potato, tobacco and *Selaginella moellendorffii* were obtained from iTAK ((http://bioinfo.bti.cornell.edu/cgi-bin/itak/index.cgi) [44] and the plant transcription factor database (http://planttfdb.cbi.pku.edu.cn/) [55]. The deduced amino acid sequences from tomato, Arabidopsis, rice, Chinese cabbage, potato, tobacco and *Selaginella moellendorffii* were aligned using the multiple alignment tool ClustalX (http://www.clustal.org/clustal2/) [56]. A phylogenetic tree was constructed using the most conserved ZF-HD dimer domains with MEGA6.0 software following the neighbor-joining (NJ) algorithm [57] with 1000 bootstrap replicates. A second phylogenetic tree of the full-length amino acid sequences of 22 tomato ZHD proteins was constructed following the UPGMA (Unweighted Pair Group Method with Arithmetic Mean) method using complete deletion of amino acids with 1000 bootstrap replicates.

### Analysis of putative cis-element in the promoter regions of *SlZHD* genes

Approximately 5 to 10 bp putative cis-elements in tomato *ZHD* genes were detected using the PlantCARE (http://bioinformatics.psb.ugent.be/webtools/plantcare/html/) [58] web-based tool. The region from the start codon of each gene to the 2000 bp upstream sequence [59] was used to identify cis-regulatory elements, because cis-elements bound by transcription factors are present in these upstream regions that regulate target genes [60].

### Chromosomal locations, gene duplication, and microsynteny analysis of tomato *ZHD* genes

The start and end positions of each tomato *ZHD* gene, including subgenome information, were obtained from SGN (https://solgenomics.net/) [43]. The position of each gene on the tomato chromosomes was analyzed using the MapGene2Chromosome2 ((http://mg2c.iask.in/) [61] web tool. A NCBI BLAST search (https://blast.ncbi.nlm.nih.gov/Blast.cgi) [50] of the tomato *ZHD* genes against each other was conducted to identify duplicated genes based on the query coverage percentage and identity of each gene. When the query coverage percentage and identity of the candidate genes was ≥80% [21], they were considered to be segmentally duplicated genes. Duplication lines among the segmentally duplicated genes were drawn manually on the chromosomes. Paralogous genes were considered to be tandemly duplicated when two genes were separated by five or fewer genes in a 100-kb region on a chromosome [22]. A BLAST search against the whole genomes of Arabidopsis, tomato, and potato was conducted to determine the microsyntenic relationships of *ZHD* genes among these species; the results were displayed using Circos software (http://circos.ca/) [62].

### Calculation of Ka/Ks ratios

The synonymous (KS) and non-synonymous (Ka) nucleotide substitution rates of *SlZHD* genes were calculated based on their coding sequence alignments following the Nei and Gojobori model with Mega 6.0 [56] software. The Ka/Ks ratios between duplicated genes were analyzed to identify the mode of selection. In general, Ka/Ks ratio > 1, <1, and =1 indicates accelerated evolution with positive selection, functional constraint with purifying selection, and neutral selection of genes, respectively [63]. Divergence time (T) of each duplicated gene pair was calculated using the formula T = *Ks*/2r Mya (Millions of years) where, *Ks* is the synonymous substitutions per site and r is considered 1.5 × 10^−8^ substitutions per site per year for dicot plants [64].

### Plant material preparation and collection

Tomato seeds of the cultivar ‘Ailsa Craig’ were germinated in potted soil in a growth room. Seed germination and seedling growth performed in a growth room with a controlled environment at 25 °C day/20 °C night temperatures, with a 16/8 h (light dark) photoperiod, 55–70% relative humidity, and 300 μmol m^−2s−1^ light intensity. Leaf, stem, and root tissues were collected from 28-day-old seedlings for organ-specific expression analysis, and the remaining seedlings were transferred to a greenhouse with at 18 ± 2 °C and 65–80% relative humidity. Flower samples were collected during full bloom at the anthesis stage. Six developmental stages (based on approximate fruit size and color) of fruits were collected: i) 1 cm, 2 weeks after pollination; ii) immature (IM), 2 cm in diameter and 20 days after pollination; iii) mature green (MG); iv) breaker stage (B), when the fruit color turned from green to yellowing-orange; v) (B + 5), 5 days after the breaker stage of fruit; and vi) (B + 10), 10 days after the breaker stage [65, 66]. Samples were collected from three biological replicates, immersed in liquid nitrogen, and stored at −80 °C for further analysis.

Potted seedlings (28 days old) were subjected to various stress treatments. Three fresh seedlings per treatment were incubated at 40 °C and 4 °C for 24 h to confer heat and cold stress, respectively. For drought stress treatment, the seedlings were removed from the soil and transferred to a dry paper towel for 24 h. NaCl stress treatment involved submerging the roots of seedlings in 200 mM NaCl for 24 h. For ABA treatment, the leaves of seedlings were sprayed with 100 μM ABA solution. Plants growing in pots under normal conditions (25 °C) were used as the 0 h controls for heat and cold, drought, NaCl, and ABA treatment. After applying different treatments, leaf samples from three biological replicates were collected at different time points (0 h, 1 h, 3 h, 9 h, and 24 h), immediately immersed in liquid nitrogen, and stored at −80 °C for RNA extraction and cDNA synthesis.

### Expression analysis of tomato *ZHD* genes

Total RNA was isolated from the plant samples using an RNeasy mini kit (Qiagen, Valencia, USA) and purified with a Qiagen RNase free DNase1 kit. RNA concentrations were measured using NanoDrop® 1000 Spectrophotometer (Wilmington, DE, USA). First-strand cDNA was synthesized using 6 ng total RNA per sample with a Superscript® III First-Strand cDNA synthesis kit (Invitrogen, Carlsbad, CA, USA). Gene-specific primers for the candidate tomato *ZHD* genes were designed using Primer3 software (http://frodo.wi.mit.edu/primer3/input.htm) [67] (Additional file 2: Table S1). The primers for *EF1a* (F: TCAGGTAAGGAACTTGAGAAGGAGCCT, R: AGTTCACTTCCCCTTCTTCTGGGCAG) from *S. lycopersicum* were used as an internal control [68]. The reaction mixture for qRT-PCR (10 μL total volume) contained 75−80 ng/μL of cDNA, 2 μL forward and reverse primers, 2 μL double distilled water, and 5 μL iTaq™ from the SYBR® Green PCR kit (California, USA). A Light cycler® 96SW 1.1 (Roche, Germany) was used for amplification and detection using the following PCR parameters: pre-denaturation at 95 °C for 5 min followed by 40 cycles of 94 °C for 10 s, annealing at 58 °C for 10 s, and extension at 72 °C for 15 s. The 2^−∆∆Ct^ method was used for data analysis [69]. Relative gene expression levels were normalized against the expression of the housekeeping gene *EF1a*. The significance of differences among relative expression levels of the genes for different samples and time points was analyzed using one-way analysis of variance (ANOVA) with MINITAB statistical software 17 (Minitab Inc., State College, PA, USA). Tukey’s pairwise comparison test was employed to determine the mean separation of expression values. The RNAseq data of *SlZHD* genes were downloaded from the Solgenomics database (https://solgenomics.net/) [47] and Tomato functional Genomic Database (http://ted.bti.cornell.edu/cgi-bin/TFGD/digital/home.cgi) [70].

## Additional files

Additional file 1: **Fig. S1.** Logos of 10 conserved motif identified by MEME software. **Fig. S2.** Chromosomal locations of *SlZHD* genes. The 22 genes are widely distributed on six of the 12 tomato chromosomes. The chromosomes number is indicated at the top of each vertical bar. The duplicated genes are connected with pink dotted line. The colored box in front of each gene indicates the subfamily according to phylogenetic tree. The scale indicates the length of the chromosome (PPTX 304 kb)

Additional file 2: **Table S1.** List of primers used for qRT-PCR analysis and their sequence, product size, primer length, primer-designing site, GC%, and melting temperature. **Table S2.** Pairwise identities and divergence between paralogous pairs of *ZHD* genes from tomato, and details about the segmental duplication of these genes. **Table S3.** Putative cis-elements >5 bp identified in 22 *SlZHD* genes from *Solanum lycopersicum* using the PlantCARE database (XLSX 43 kb)

Additional file 3: **Table S1.** Information of *ZHD* genes of Putative tomato, Arabidopsis, potato, tobacco, chinese cabbage, rice and *Selaginella moellendorffii* used in in silico analysis. **Table S2.** Online RNA sequencing data (PKRM) downloaded from Solgenomics database (https://solgenomics.net/) and Tomato functional Genomic Database (http://ted.bti.cornell.edu/cgi-bin/TFGD/digital/home.cgi) (XLSX 35 kb)

Additional file 4: **Fig. S1.** Multiple sequence alignment of the conserved domain of ZHD protein family of tomato, potato, tobacco, Arabidopsis, chinese cabbage, rice and *Selaginella moellendorffii,* in where black and grey shading indicating 100% and 60% identity, respectively. **Fig. S2.** Phylogenetic relationship of Arabidopsis(AtZHD), rice(OsZHD), potato (St, *Solanum tuberosum* is used instead of PGSC0003DMT4000), tobacco (Nt, *Nicotiana tabacum* is used instead of XP_0164), Chinese cabbage (BraZF-HD), *Selaginella moellendorffii*, (SmZF-HD) and tomato (SlZHD) ZHD proteins. The conserved ZF-HD_ dimer domain sequences of Arabidopsis, rice, potato, tobacco, Chinese cabbage, *Selaginella moellendorffii*, and tomato proteins were aligned using ClustalX, and the tree were constructed by the Maximum likelihood method with MEGA 6.0. The numbers on the branches indicate bootstrap support values from 1000 replications. The protein sequences used in the phylogenetic analysis are listed in Additional file 1, along with their accession numbers. The tree was divided into six subfamilies according to bootstrap support values and evolutionary distances (PPTX 818 kb)

## Abbreviations

aa: amino acid
ABA: Abscisic acid
*At*: *Arabidopsis thaliana*
bp: base pair
BRAD: *Brassica* database
Ka: Nonsynonymous substitutions per nonsynonymous site
kDa: Kilo Dalton
Ks: Synonymous substitutions per synonymous site
MW: Molecular weight
MYA: Million year
ORF: Open reading frame
pI: isoelectric points
qRT-PCR: Quantitative polymerase chain reaction
Sl: *Solanum lycopersicum*
*Sm*: *Selaginella moellendorffii*
TIGR: Rice Genome Annotation Project
ZHD: Zinc finger homeodomain
