# Supplementary material for: Genome-wide analysis and expression profiling of zinc finger homeodomain (ZHD) family genes reveal likely roles in organ development and stress responses in tomato
Source: BMC Genomics. 2017 Sep 6;18:695. doi: 10.1186/s12864-017-4082-y (PMC5585987; doi:10.1186/s12864-017-4082-y)
Supplement: Supplementary file 1 — Fig. S1. Logos of 10 conserved motif identified by MEME software. Fig. S2. Chromosomal locations of SlZHD genes. The 22 genes are widely distributed on six of the 12 tomato chromosomes. The chromosomes number is indicated at the top of each vertical bar. The duplicated genes are connected with pink dotted line. The colored box in front of each gene indicates the subfamily according to phylogenetic tree. The scale indicates the length of the chromosome (PPTX 304 kb) [file 12864_2017_4082_MOESM1_ESM.pptx]

## Slide 1
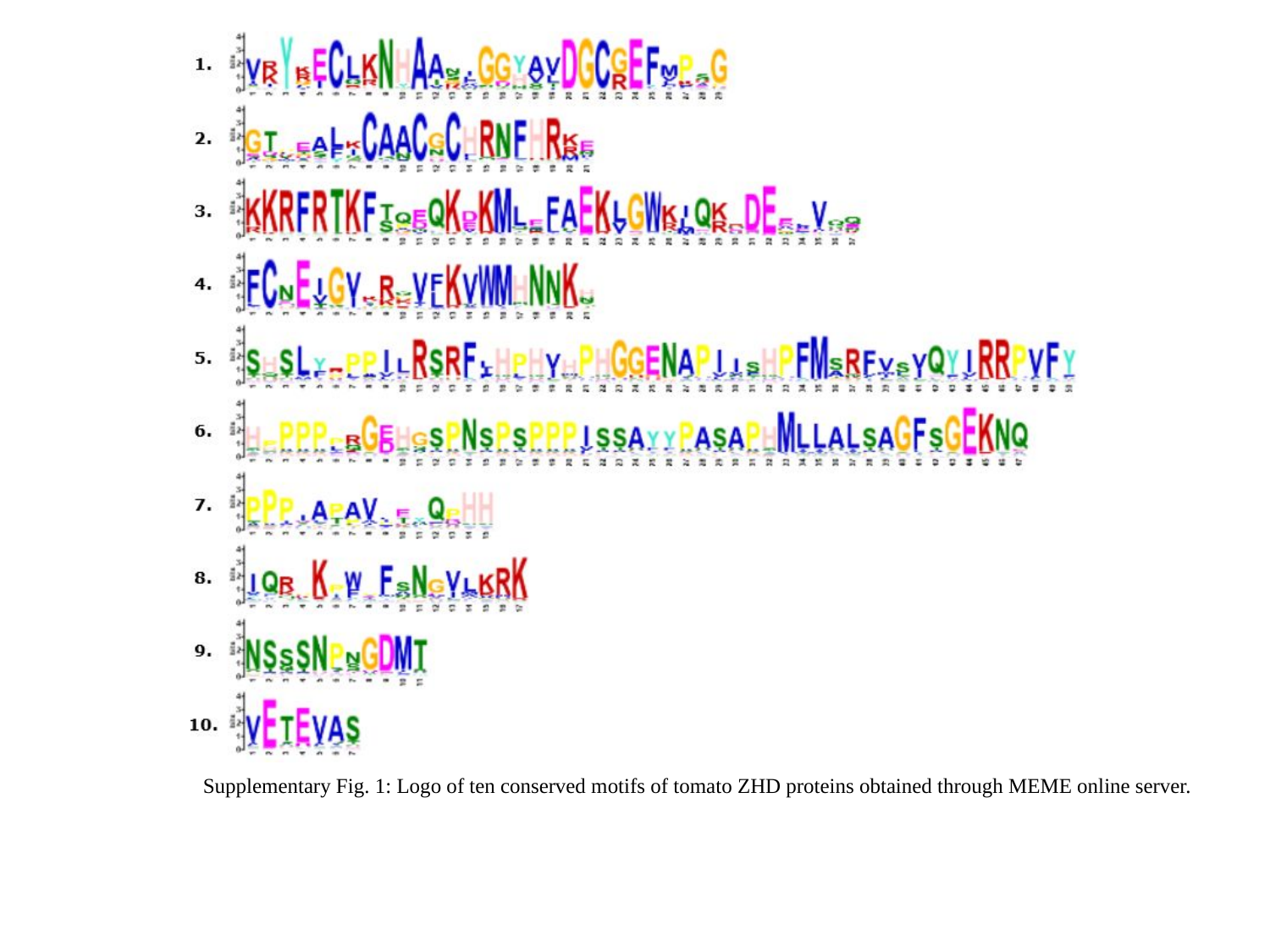

Supplementary Fig. 1: Logo of ten conserved motifs of tomato ZHD proteins obtained through MEME online server.

## Slide 2
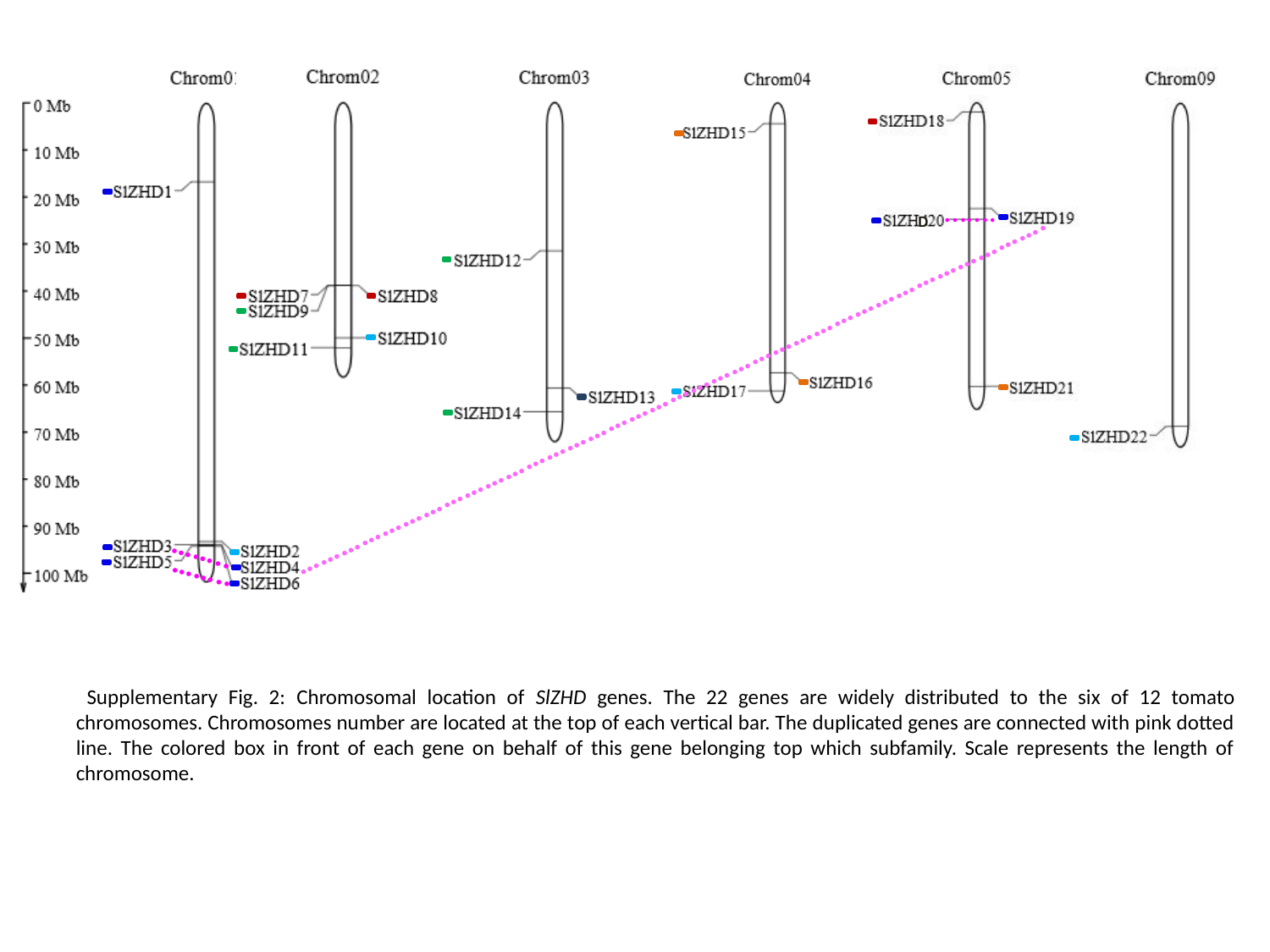

…….
D
……………………………………………………………………………………………….
……..
……..
 Supplementary Fig. 2: Chromosomal location of SlZHD genes. The 22 genes are widely distributed to the six of 12 tomato chromosomes. Chromosomes number are located at the top of each vertical bar. The duplicated genes are connected with pink dotted line. The colored box in front of each gene on behalf of this gene belonging top which subfamily. Scale represents the length of chromosome.
