# Supplementary material for: Genome-wide analysis and expression profiling of zinc finger homeodomain (ZHD) family genes reveal likely roles in organ development and stress responses in tomato
Source: BMC Genomics. 2017 Sep 6;18:695. doi: 10.1186/s12864-017-4082-y (PMC5585987; doi:10.1186/s12864-017-4082-y)
Supplement: Supplementary file 4 — Fig. S1. Multiple sequence alignment of the conserved domain of ZHD protein family of tomato, potato, tobacco, Arabidopsis, chinese cabbage, rice and Selaginella moellendorffii, in where black and grey shading indicating 100% and 60% identity, respectively. Fig. S2. Phylogenetic relationship of Arabidopsis(AtZHD), rice(OsZHD), potato (St, Solanum tuberosum is used instead of PGSC0003DMT4000), tobacco (Nt, Nicotiana tabacum is used instead of XP_0164), Chinese cabbage (BraZF-HD), Selaginella moellendorffii, (SmZF-HD) and tomato (SlZHD) ZHD proteins. The conserved ZF-HD_ dimer domain sequences of Arabidopsis, rice, potato, tobacco, Chinese cabbage, Selaginella moellendorffii, and tomato proteins were aligned using ClustalX, and the tree were constructed by the Maximum likelihood method with MEGA 6.0. The numbers on the branches indicate bootstrap support values from 1000 replications. The protein sequences used in the phylogenetic analysis are listed in Additional file 1, along with their accession numbers. The tree was divided into six subfamilies according to bootstrap support values and evolutionary distances (PPTX 818 kb) [file 12864_2017_4082_MOESM4_ESM.pptx]

## Slide 1
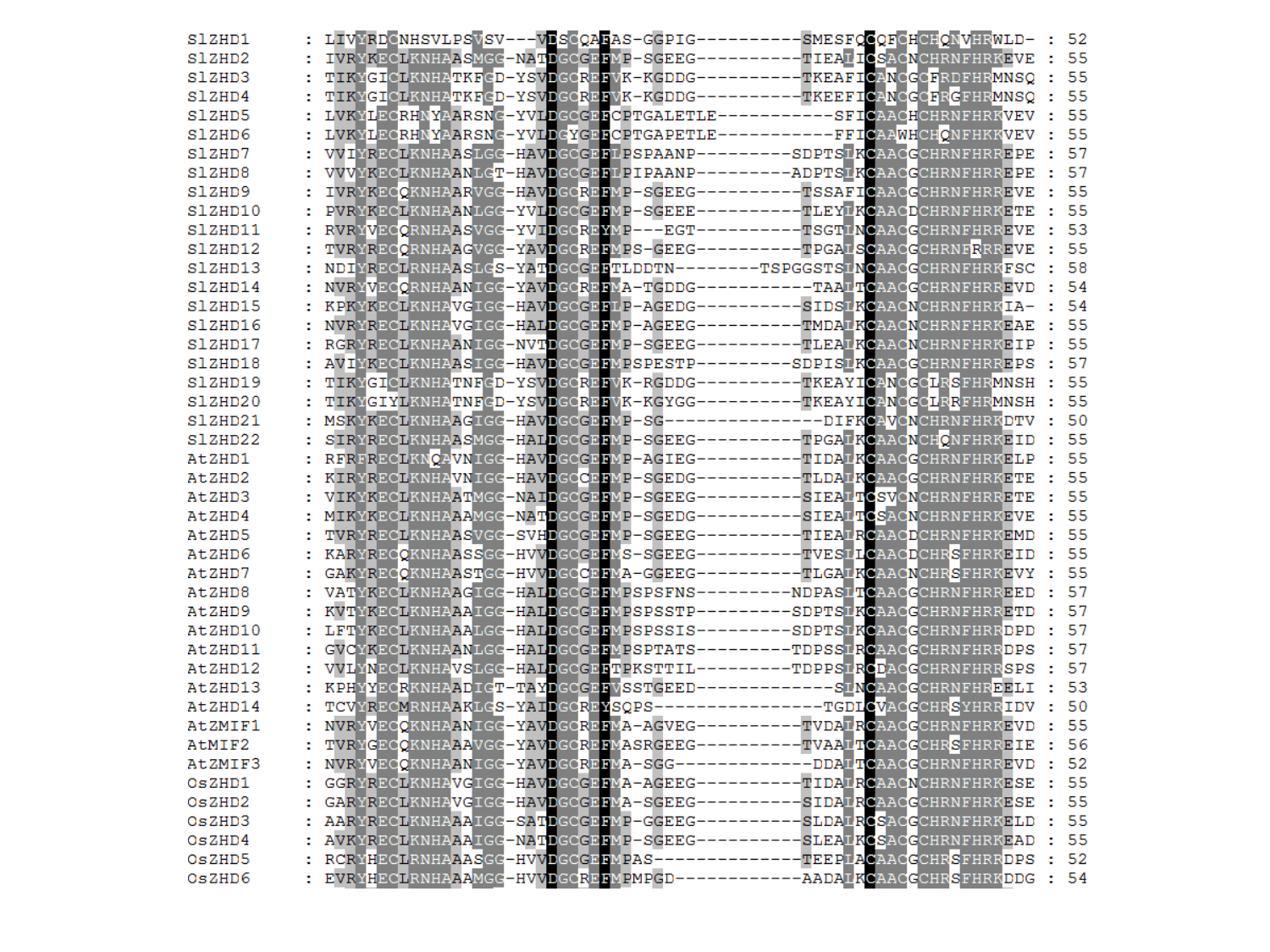

## Slide 2
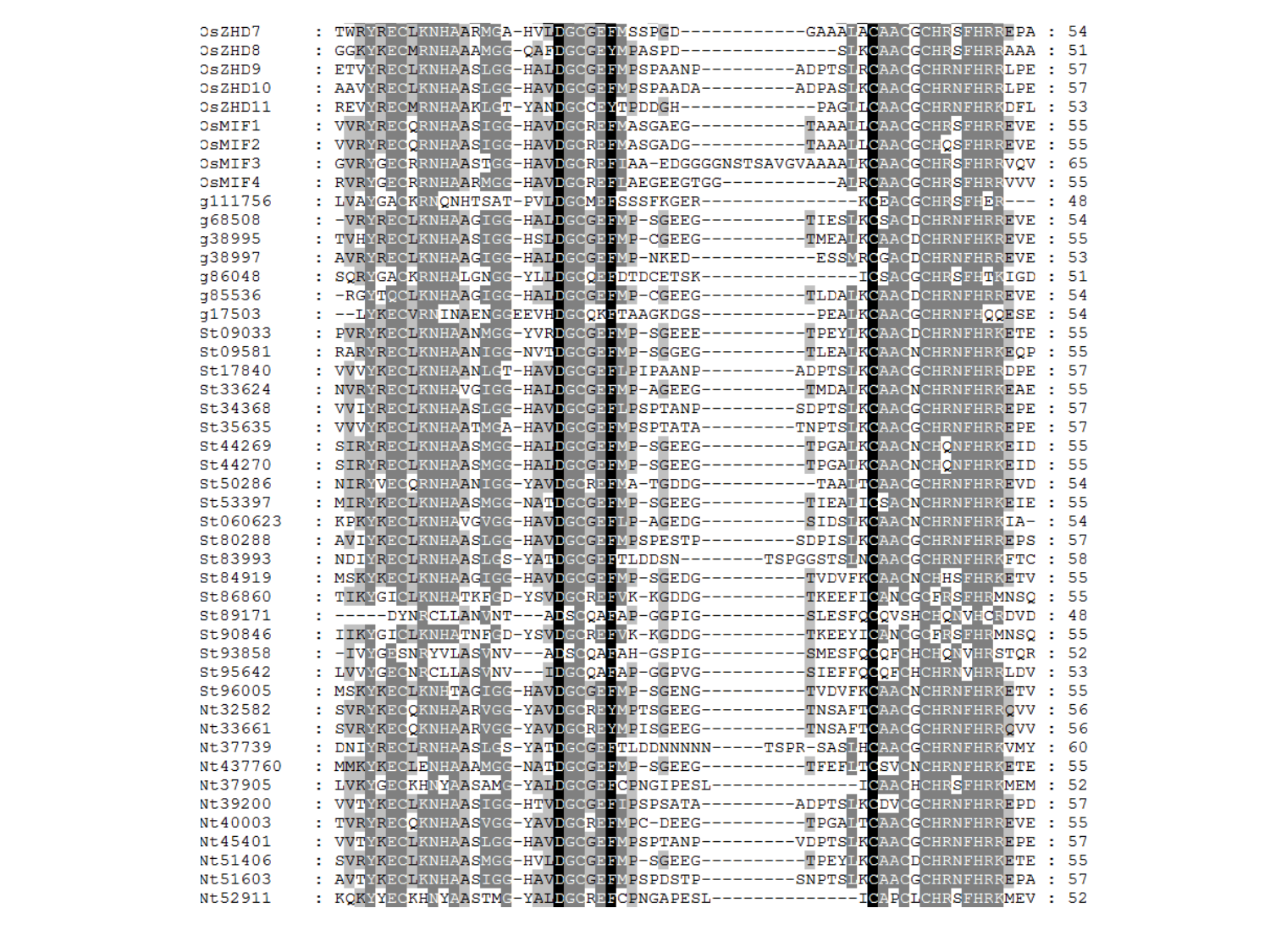

## Slide 3
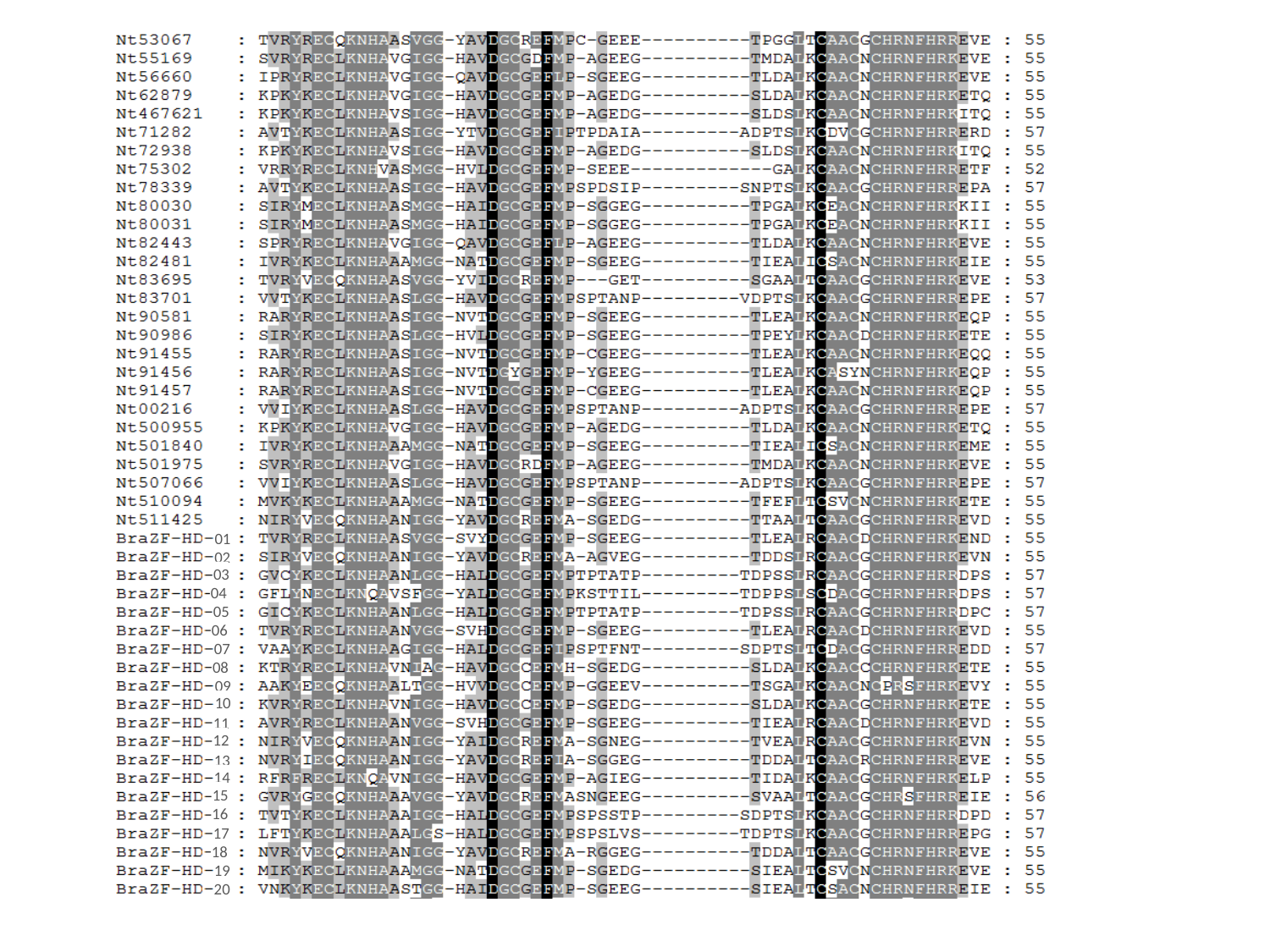

01
02
03
04
05
06
07
08
09
10
11
12
13
14
15
16
17
18
19
20

## Slide 4
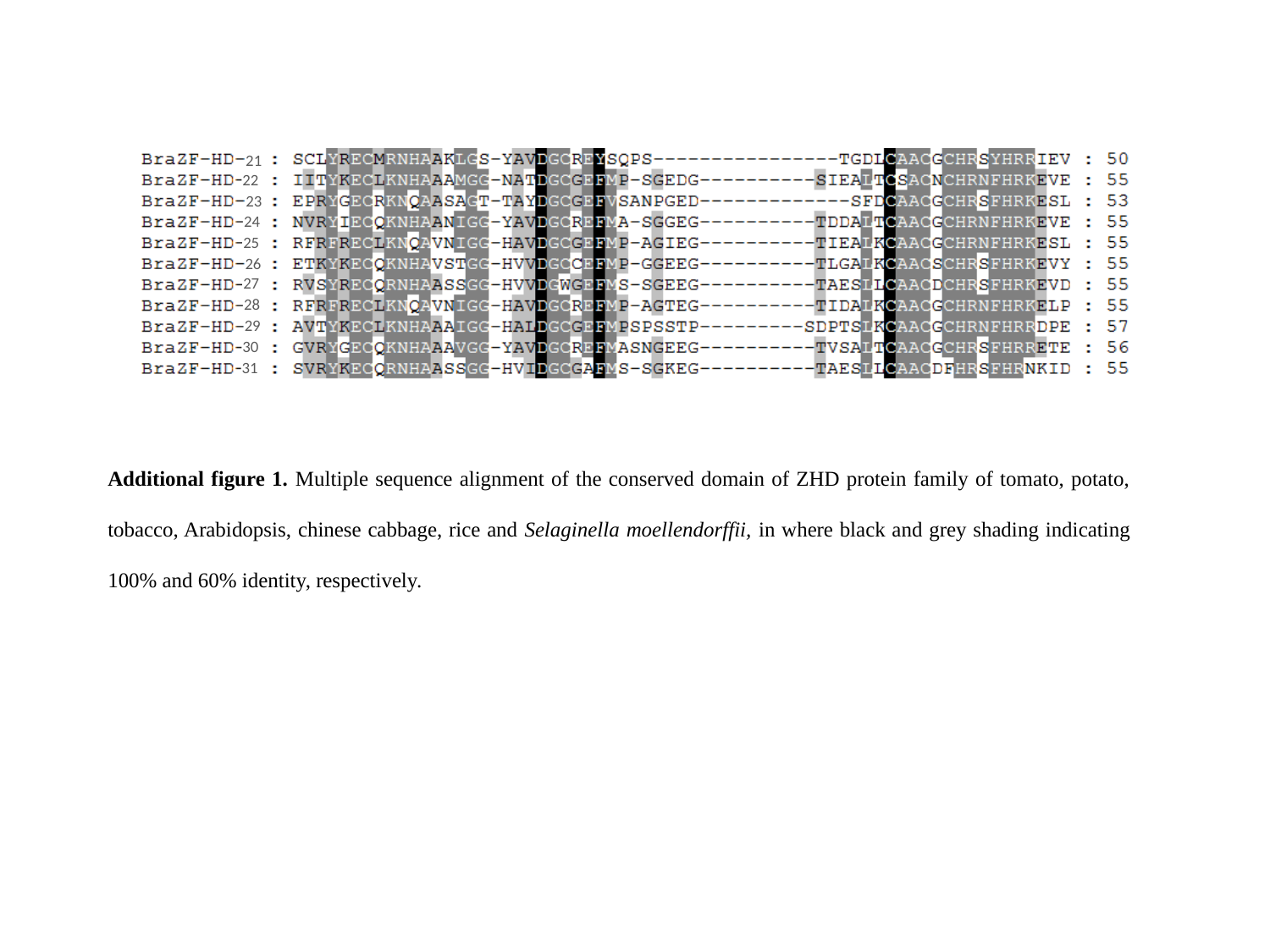

21
22
23
24
25
26
27
28
29
30
31
Additional figure 1. Multiple sequence alignment of the conserved domain of ZHD protein family of tomato, potato, tobacco, Arabidopsis, chinese cabbage, rice and Selaginella moellendorffii, in where black and grey shading indicating 100% and 60% identity, respectively.

## Slide 5
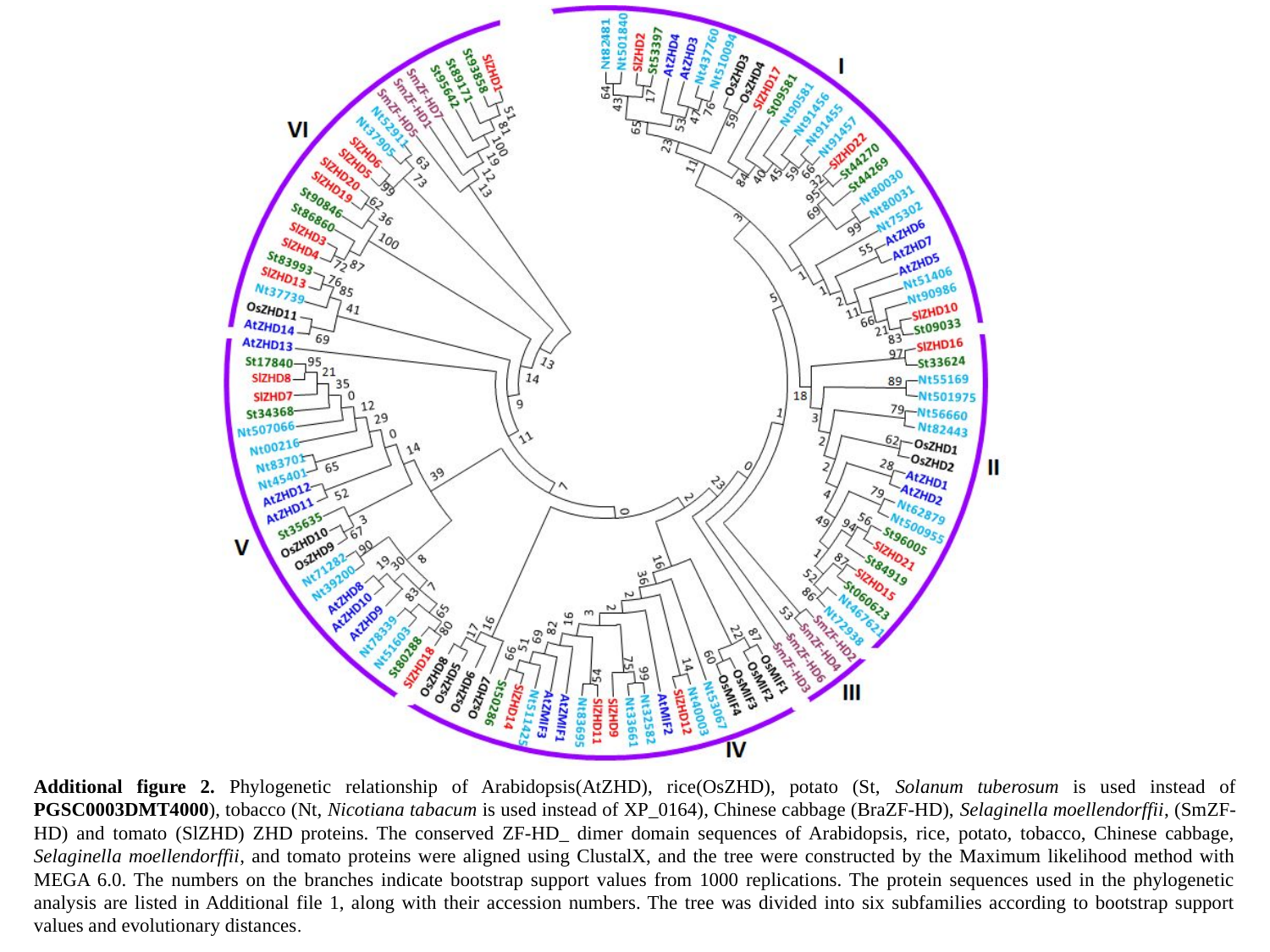

Additional figure 2. Phylogenetic relationship of Arabidopsis(AtZHD), rice(OsZHD), potato (St, Solanum tuberosum is used instead of PGSC0003DMT4000), tobacco (Nt, Nicotiana tabacum is used instead of XP_0164), Chinese cabbage (BraZF-HD), Selaginella moellendorffii, (SmZF-HD) and tomato (SlZHD) ZHD proteins. The conserved ZF-HD_ dimer domain sequences of Arabidopsis, rice, potato, tobacco, Chinese cabbage, Selaginella moellendorffii, and tomato proteins were aligned using ClustalX, and the tree were constructed by the Maximum likelihood method with MEGA 6.0. The numbers on the branches indicate bootstrap support values from 1000 replications. The protein sequences used in the phylogenetic analysis are listed in Additional file 1, along with their accession numbers. The tree was divided into six subfamilies according to bootstrap support values and evolutionary distances.
